# Ice thickness monitoring for cryo-EM grids by interferometry imaging

**DOI:** 10.1038/s41598-022-16978-7

**Published:** 2022-09-12

**Authors:** Markus Matthias Hohle, Katja Lammens, Fabian Gut, Bingzhi Wang, Sophia Kahler, Kathrin Kugler, Michael Till, Roland Beckmann, Karl-Peter Hopfner, Christophe Jung

**Affiliations:** 1 Gene Center and Department of Biochemistry, (CIPSM), Ludwig-Maximilians-Universität München, Feodor-Lynen-Strasse 25, 81377 München, Germany; 2 MathWorks, Ismaning, Germany

**Keywords:** Cryoelectron microscopy, Interference microscopy

## Abstract

While recent technological developments contributed to breakthrough advances in single particle cryo-electron microscopy (cryo-EM), sample preparation remains a significant bottleneck for the structure determination of macromolecular complexes. A critical time factor is sample optimization that requires the use of an electron microscope to screen grids prepared under different conditions to achieve the ideal vitreous ice thickness containing the particles. Evaluating sample quality requires access to cryo-electron microscopes and a strong expertise in EM. To facilitate and accelerate the selection procedure of probes suitable for high-resolution cryo-EM, we devised a method to assess the vitreous ice layer thickness of sample coated grids. The experimental setup comprises an optical interferometric microscope equipped with a cryogenic stage and image analysis software based on artificial neural networks (ANN) for an unbiased sample selection. We present and validate this approach for different protein complexes and grid types, and demonstrate its performance for the assessment of ice quality. This technique is moderate in cost and can be easily performed on a laboratory bench. We expect that its throughput and its versatility will contribute to facilitate the sample optimization process for structural biologists.

## Introduction

Over the last decades cryo-EM has proven to be a powerful approach for the structure determination of proteins and biological complexes, now routinely delivering structures at near-atomic resolution^1–4^ The method is based on imaging randomly oriented single particles embedded in thin vitreous ice with an electron microscope, followed by image processing and 3D reconstruction of the macromolecular complex. However, in cryo-EM the procedure leading from purified sample to the final reconstructed biological structure is complex and time consuming, mainly due to the computationally intensive data processing, but also due to challenging sample preparation. A particularly critical experimental factor is the thickness of the ice layer in which the particles of interest are commonly embedded: the particles would not fit or could be distorted in a too thin film compared to their size, whereas image contrast would be severely impaired with a too thick ice layer because of electron absorption. Hence, for every sample there is an optimal ice thickness that ideally should be just thick enough to support all orientations of the particles, which roughly corresponds to the particle size. Samples are commonly prepared by blotting an aqueous solution containing the biological material (usually a purified protein or protein/nucleic acid complex) onto a grid support (often in copper or gold), followed by plunge freezing into liquid ethane or propane, fast enough to prevent ice formation^5–7^. In practice, for a given protein or complex an experimenter prepares a series of samples using various conditions for buffer composition, additives like detergents, blotting time and strength, grid type and glow discharge conditions. This leads to a concentration range of protein embedded in an ice layer with varying thicknesses and uniformity across the support structure. Usually the grids are then imaged at low-magnification using an electron microscope. Since a thicker ice layer transmits fewer electrons than a thinner film, the user is then able to select suitable samples based on the brightness of the images inside the holes of the grids, for which high-resolution cryo-EM can be performed. This procedure has, however, limitations. First, the visual assessment of ice quality from the EM images requires much experience, and it is therefore strongly dependent on the experimenter. In addition, it is necessary to image each of the coated grids for quality assessment, which is time consuming with an electron microscope. Here, the loading of the sample within the measurement chamber and the image acquisition are relatively slow processes. Even with automated sample loading and acquisition the screening of 12 grids requires a day of measurement time. In addition, electron microscope usage is expensive and the machines are usually extensively booked. Thus, this procedure constitutes a significant rate-limiting step in cryo-EM, especially for new users with limited access to measurement time at facilities.

We reason that sample quality diagnostic -in particular regarding the ice thickness and its uniformity across the grid- is a bottleneck that could substantially benefit from a faster imaging method. Over the past years attempts were made to develop routine methods for grid characterization by tomography^8^ or using an energy filter or scattering outside the objective aperture^9^ to measure ice thickness. Other publications present aperture-limited scattering (ALS)-based methods^10–12^ or a deep-learning based algorithm^13^ to facilitate researchers to select ‘good’ regions with appropriate ice thickness for imaging. Another study analyses directly the grey values distribution of foil-hole images to infer ice quality^14^. However, although these techniques can help for a diagnostic on amorphous ice quality, they still use images acquired on standard electron microscopes, and are thus relatively slow. One method selects images with appropriate ice thickness only after complete high resolution EM imaging of a sample^15^. As a consequence, all these approaches do not substantially accelerate the sample screening procedure. Thus, it would be highly desirable to have a faster technique available using an easier-to-use microscope for grid quality assessment. Ideally, the instrument should also be provided with a software pipeline for the automated recognition of good sample regions, which would greatly help the user for sample selection.

The determination of amorphous ice thickness proves to be difficult with most conventional techniques for film thickness measurements like spectroscopy and ellipsometry since they are generally single-point measuring methods and suffer from low spatial resolution^16^. An appealing solution for the two-dimensional thickness measurement with a relatively simple setup design is to illuminate a larger region of the sample with three-wavelengths simultaneously, and to capture the interference patterns produced by the sample with a colour camera^17,18^. The film thickness can be estimated at each pixel after calibration of the setup. However, the colour-thickness relationship depends on many parameters, in particular on the nature of the sample itself, which makes the calibration procedure difficult. In addition, the use of complex algorithms is required to achieve thickness determination with nano-scale z-height resolution. Similar drawbacks apply to other 2D-measurement based approaches like spectrophotometric-^19,20^, ellipsometric-^21^ or white-light interferometry-imaging^16^. Furthermore, these techniques were developed for the thickness determination of typically thin inorganic films such as polymers or semiconductors. To our knowledge they were not yet adapted for an ice layer at cryogenic temperature.

**Figure 1:**
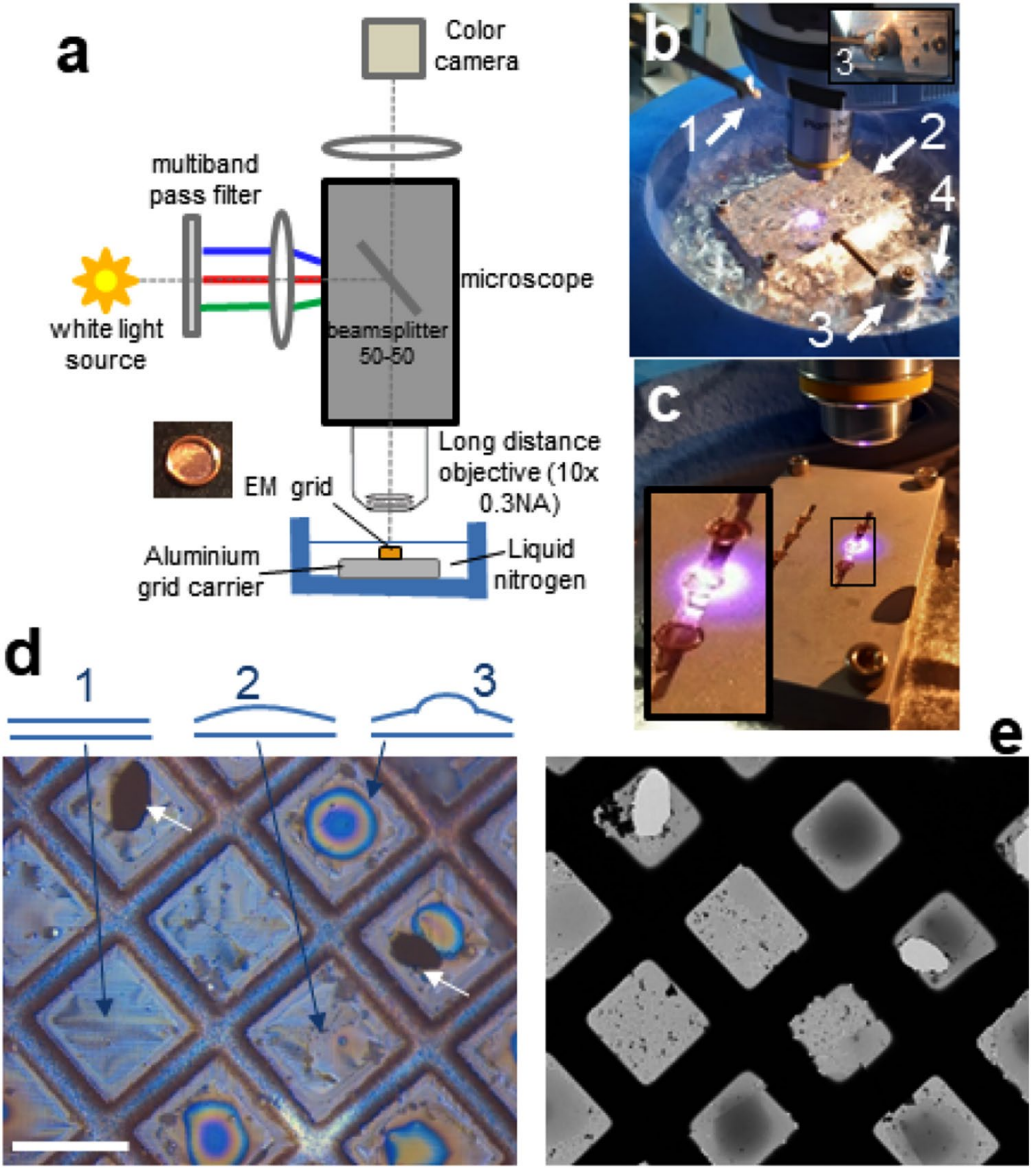
Experimental setup for interferometric microscopy at cryogenic temperature. (**a**) Optical setup. The insert shows an exemplary EM grid that will be coated with a vitrified ice layer containing the protein complex. (**b**) Image of the custom-made cryo-stage filled with liquid nitrogen, also showing the tweezer holding the grid coated with sample (arrow 1), the aluminium heat sink supporting the grid (arrow 2), the grid-box transfer mount (arrow 3, magnification from another view in the top right corner), and a grid-box (arrow 4). (**c**) Picture of the aluminium heat sink on which grids are placed for imaging. The insert shows a magnification showing three grids, the middle one being illuminated by the three-wavelength light through the microscope objective. (**d**) Top: schematic diagrams of grid hole cross-sections of (1) ideal ice thickness where the particles tightly fit, (2) region with thicker ice regions in which particle density is too high for optimal cryo-EM imaging, and (3) too thick ice with the presence of water droplets not suitable for the measurements. Bottom: typical interferometric image of a grid coated with an ice layer containing the sample. The interference colour patterns reveal the presence of the characteristic regions described on top (three different cases indicated by the arrows). Scale bar 50 µm. (**e**) Corresponding low-magnification EM image of the same grid region. The EM user usually estimates ice thickness based on the brightness of the images inside the holes of the grids since a thicker ice layer transmits less electrons than a thinner film. The comparison with the interferometric in (**d**) confirms the presence of different ice layer thicknesses (top in **d**), as identified by the coloured interference patterns.

**Figure 2:**
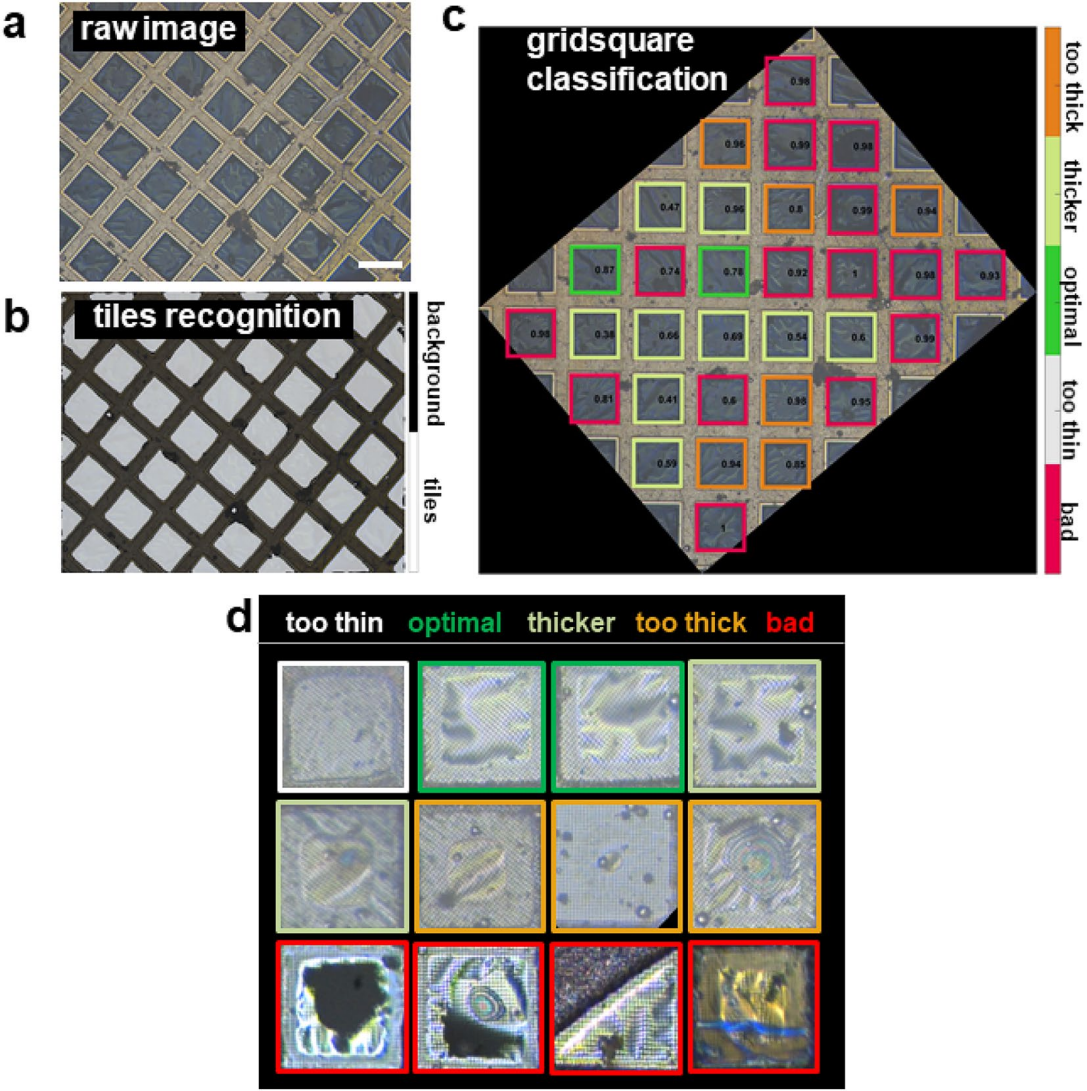
ANN analysis of the interferometric images for the determination of ice quality. (**a**) Raw interferometric image of a copper grid coated with sample prior analysis by ANNICAS. Scale bar 50 µm. (**b**) The same image overlaid with the tiles (in grey) as recognized by the segmentation network after complete training (see main text and Methods). All tiles were correctly identified. (**c**) The same image as in (**b**) after full analysis and grid square classification with the fully trained classification network. The image was rotated during analysis. The grid squares are highlighted with colours according to the ice layer quality. Five classes for grid squares quality were defined: bad (class 0, in red), too thin (class 1, in white), optimal (class 2, in green), thicker (class 3, in bright green) and too thick (class 4, in orange), respectively (colour legend indicated on the right). Numbers indicate confidence of ANN. (**d**) Examples of grid square classification for a wide spectrum of different interferometric images. Ice thickness of grid squares ranged from ‘too thin’ (in white) to too thick (in orange), with also grid squares with ice thickness in between (in green or light green), or with grid squares presenting large defects (in red). Colour-code for ice quality shown on the top.

To remedy this situation, we devised a method that combines an optical interferometric microscope at cryogenic temperature (Fig. 1) with an image analysis software that can assess whether the samples are suitable for subsequent cryo-EM measurement (Fig. 2). The experimental setup comprises a conventional optical microscope adapted for reflected interferometric imaging, fitted with a cryogenic stage (Fig. 1a–c and Methods). The image analysis is based on artificial neural networks (ANN), a deep-learning approach that successfully addressed a wide range of complex image analysis problems^22–25^. Our software automatically detects the grid squares visible in an interferometric image, and classifies them according to ice quality. Hence, this configuration allows for fast and convenient batch-screening of the ice thickness on EM grids, followed by the automated recognition of sample regions with an appropriate ice layer. Herein, we present this method and demonstrate its benefits for the selection of sample grids potentially suitable for high-resolution cryo-EM.

## Results

### Interferometric microscopy setup

In the instrument (Fig. 1a), an EM grid supporting the protein (arrow 1 in Fig. 1b) is placed on an aluminium carrier (arrow 2 and Fig. 1c) in a custom-made cryogenic stage (the blue container in Fig. 1b) filled with liquid nitrogen. A grid-box transfer mount (arrow 3 in Fig. 1b) allows the immobilization of the cryo-grid box for the grid transfer to the aluminium carrier (the latter can accommodate multiple grids). A given grid is illuminated by three different wavelengths simultaneously using a conventional widefield microscope that is equipped with long working distance dry objectives with different magnifications (5x, 10 × and 20x), a 50/50 beamsplitter, and a sensitive colour camera (Fig. 1a and Methods). These modifications are readily implemented and moderate in cost. To test our system, we chose the bacterial DNA double-strand break sensor SbcCD from *E.coli* bound to a 120 bp DNA oligomer^26^. In addition, we used the same DNA blocked with the Ku70/80 protein at the DNA end in complex with SbcCD bound to the non-hydrolysable ATP analog ADP-BeF for further investigation (manuscript in preparation). The SbcCD complex exhibits an intriguing 40-nm-long coiled coil(CCs) region, with a moderate molecular weight of the full heterotetrameric complex of 326 kDa^26^. These features make these particles ideal test objects since they are challenging to image by cryo-EM due to their complex shape with a relatively small molecular size. A further complication in terms of sample preparation is that the Ku70/80 complex tends to cluster and fall apart in the presence of detergents, which prevents optimization of amorphous ice layer quality by, e.g,. β -octylglucoside. The SbcCD sample was applied on glow-discharged grids (Methods) that were then readily transferred (manually with a tweezer designed for EM, arrow 1 in Fig. 1b) from their cryo-grid box (arrow 4 in Fig. 1b) to the aluminium carrier, constantly kept under liquid nitrogen to preserve the sample. Typically, the whole procedure including manipulating the grids, setting the focus and imaging multiple regions for each sample takes less than ~ 15 min for 4 grids, the content of a grid-box. After data collection, the grids are transferred back to their grid-box, that will be stored at cryogenic temperature for possible subsequent imaging in the electron microscope. We checked that this experimental procedure does not damage the ice layer, provided that usual precautions with the coated grids are met. This procedure can apply for standard grids but also for so called clipped grids prepared for automated sample transfer into a state-of-the art cryo-EM (Titan, Glacios, Arctica, etc..).

A typical image collected after light reflection on a grid (Fig. 1d) exhibits distinct interference patterns produced by the different excitation colours reflected by the ice layers. An immediate observation is the strong heterogeneity between and within the individual grid square patterns (visible as squares in Fig. 1d). This reflects the varying ice thickness across the grid, a phenomenon commonly observed in cryo-EM. The comparison with the low magnification EM image of the same region (Fig. 1e) confirms the presence of regions with thicker vitreous ice revealed by darker patterns in the middle of several grid squares. Surprisingly, if the optical image shows globally similar features compared to the EM images, a closer inspection reveals subtle differences. For example, many more fine structures can be observed in the grid squares of the optical images, that probably arise from the not perfectly flat surfaces of the grid support. We also observe interference patterns when imaging control grids without coating of sample (example in Supplementary Fig. 6). As expected, defects like holes in the grid squares (white arrows in Fig. 1d) are well visible in both images. Overall, the grid squares can be grouped into three characteristic interference patterns: (i) relatively homogenously coloured grid squares (blue arrow 1 in Fig. 1d) with a thin vitreous ice layer (as assessed by an experienced user from Fig. 1e). These regions are potentially well suitable for subsequent high-resolution cryo-EM; (ii) grid squares exhibiting more colour variations with brown-to-reddish regions (blue arrow 2 in Fig. 1d), revealing the presence of thicker vitreous ice. This group with more inhomogeneous ice thickness is an intermediate case which, however, may be at least partially suitable for high-resolution cryo-EM in certain regions of the grid squares; (iii) regions with circular and periodic colour pattern in the centre (blue arrow 3) corresponding to very thick (in the order of the wavelength i.e. ~ 0.5 µm) vitreous ice, most probably sample droplets. The strong absorbance of the electrons well visible in the EM image as darker patterns confirm that these regions are too thick for cryo-EM imaging. Thus, the interferometric images of the grid contain valuable information about vitreous ice thickness and homogeneity.

Note that with the optical spatial resolution of the instrument (in the order of µm), the holes in the grid squares (the regions that will be actually imaged in cryo-EM) can only be barely resolved. This is however not a limitation since this resolution is sufficient to assess ice quality across the whole grid squares, thus also in the individual holes. With our setup, any type of grid with various grid square sizes and made of different material can be completely imaged (as shown in Supplementary Fig. 1 for a gold grid). In practice, however, acquiring only a few images (typically 3–4 are enough) of random regions is representative for a given grid.

### ANN-based image analysis pipeline

Although the visual inspection of the raw interferometric images is a priori sufficient for a rough diagnostic of ice quality, we seek a more quantitative image analysis pipeline, which would be faster and user independent (usually, in cryo-EM the identification of the ‘good’ regions of the grids still largely depends on the user’s experience and skills). Our goal was to provide a user-friendly software package that performs all steps from feature detection, to categorization and amorphous ice thickness analysis in a fully automated fashion. This way, the user only needs to pick the image (or chooses to run the analysis automatically while the microscope generates the images) without any basic knowledge or experience in image analysis. Given the complexity of this problem and the different parameters (grid type, image resolution, tilting angle of the grid squares, grid defects and artefacts, interferometric patterns etc..), the use of conventional image analysis tools is challenging, especially for the ice quality assessment. Thus, we are employing an ANN which is a promising approach since well-trained networks can efficiently deal with a huge number of parameters and features to solve such complex problems^27,28^ (Fig. 2).

The image analysis requires two steps (Fig. 2 and Supplementary Fig. 2): first, the square like objects (we are referring to as “tiles” from now on, Fig. 2a) of the images are detected (segmentation, Fig. 2b). Secondly, once the tiles are detected, they are classified concerning their quality (thickness of the vitreous ice layer, presence of dust or defects etc., Fig. 2c–d). To this end, the neuronal networks implemented in our software has to be trained prior to function.

As segmentation requires a different network architecture than classification, we trained two different networks. The segmentation network was trained for all three objective magnifications used and identifies the tiles robustly (Supplementary Fig. 3a). For user-friendliness, the software also rotates the image (having an arbitrary orientation) into the correct angle using a quick radon transformation^29,30^. For the classification, we defined five classes for grid squares quality: ‘bad’ (class 0), ‘too thin’ (1), ‘optimal’ (2), ‘thicker’ (3) and ‘too thick’ (4), respectively. Class 0 corresponds to grid squares exhibiting defects such as holes, scratches, dust or small amorphous ice particles. An expert user manually labelled ~4000 grid squares’ interferometric patterns from the training images according to these classes of interferometric patterns. We tested all 17 publicly available pre-trained ANNs (available in *MATLAB*^®^, Supplementary Fig. 3b). *Darknet19* (stochastic gradient descent method^31^) exhibited the best performance with a validation accuracy of 98% (Supplementary Fig. 4a) across the different classes. Supplementary Fig. 4b illustrates the low cross-entropy values by showing the histograms of the probability for each detected class, as assigned by the ANN. An example after the full analysis (detection and classification) for different grids can be seen in Fig. 2c and 4.

Once the networks are sufficiently well trained, they can be saved and incorporated in to an executable file that can be ran easily by any user, without any knowledge required about ANNs or the training process. While the training process (that ideally has to be done only once) with only a few hundred images may take many hours, the analysis, hence the actual application for the user, takes only a few seconds per image on a standard personal computer. Most conveniently, the software named ANN-based Image Categorization and Analysis Software (ANNICAS) runs in the background (costing only negligible computing time). ANNICAS automatically detects a new image in a target folder (be it if generated by the microscope or copied into the folder by a user), analyses it, and saves the analysed image into a second folder. The core of our software (available for download at https://github.com/GeneCenterMunich/ANNICAS) is written in *MATLAB*^®^ and we compiled it to a stand-alone executable for Windows, such that no MATLAB® license is required by the user. The program also automatically prepares the image for parallel processing and allocates corresponding GPUs if available in order to reduce computational time.

### Experimental validation of the ANN-based analysis

**Figure 3:**
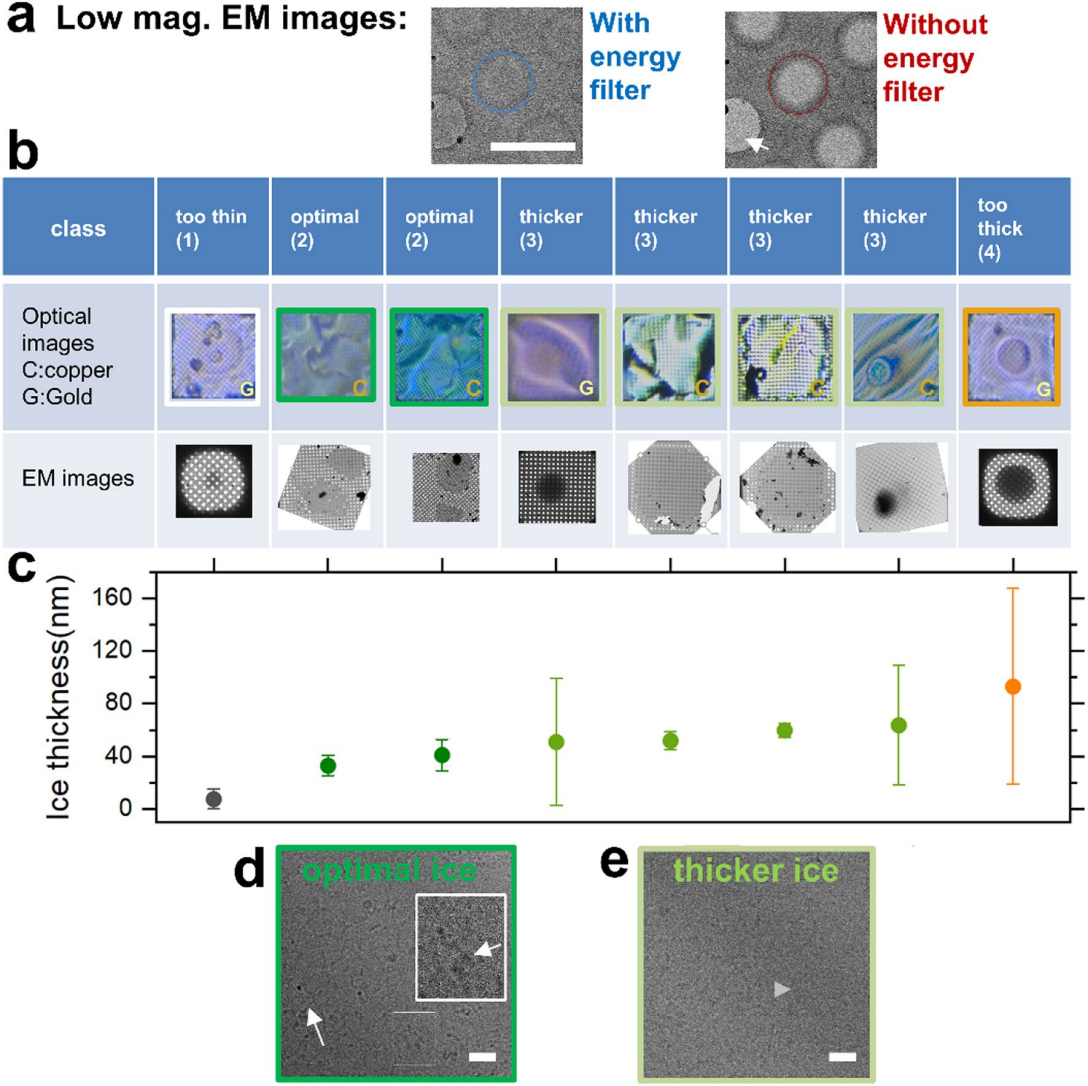
Validation using EM for the ice thickness determination. (**a**) Absolute ice thickness measurement using EM. Low magnification EM images showing holes in a grid square, and acquired with (left image) and without (right image) the activation of an energy filter. The local absolute ice thickness can be determined from the different intensities in given holes (blue and red circles) (Methods). (**b**) Correlation between the ANNICAS grid square classification and the ice layers measurements obtained by EM. Higher row ‘Optical images’: optical interferometric images for eight representative grid squares classified by ANNICAS. Middle row ‘EM images’: corresponding low-magnification EM images (if necessary after image rotation). (**b**) Representative ice thicknesses for these grid square (samples in the same order than in (**c**)) obtained by calculation of the average ice thickness (Methods) in 10 holes randomly distributed. The standard deviations of the thickness values for the 10 positions are indicated as bars. The standard deviations are large for many samples, reflecting the strong inhomogeneity of their respective ice layers. The ice thicknesses are consistent with our classification, confirming that our ANN-based analysis reliably detects and classifies the grid squares according to the ice thickness/quality. (**d–e**) High-resolution cryo-EM images in a class 2 (**d**), and in a class 4 (**e**) grid squares. SbcCD particles are distinctly visible in the left insert (white arrows), whereas the particles in too thick ice on the right are much more crowded, partially forming aggregates (grey arrow), and appear in addition with a lower contrast. Scale bars 20 nms.

To validate our classification and to evaluate quantitatively the relationship between grid square class and the actual thickness of the vitreous ice layer, we measured the local amorphous ice thickness on different copper and gold grids directly with an electron microscope (Fig. 3). This can be readily achieved if the latter is equipped with an energy filter by the direct pixel-wise comparison of filtered and unfiltered image intensities^32^ (Fig. 3a and Methods). First, we acquired interferometric images of 10 different grid squares and applied our ANN analysis for classification (‘Optical images’ row in Fig. 3b). We then acquired low-magnification EM images of the same grid squares (‘EM images’ row in Fig. 3b) that we were able to localize after the acquisition of an atlas of images of the grid (Methods). As our classification based on optical images applyies on the whole grid square rather than on the individual holes, we determined a representative vitreous ice thickness for the EM images for a given grid square by calculating the average ice thickness (Methods) in 10 randomly holes distributed (Fig. 3c). The mean thicknesses ranged from 6 to 91 nm, and increased with the class number from 1 (‘too thin’) to 4 (‘too thick’), validating our approach. The thickness variabilities, as indicated by the high standard deviations values in many cases (Fig. 3c), reflect a broad thickness distribution of the vitreous ice across all grid squares. Remarkably, the average thickness of class 2 (labelled as ‘optimal’ since its thickness can fit a wide range of protein complexes typically investigated) is perfectly consistent with 40 nm-sized CCs of SbcCD chosen as protein example herein. Hence, the experimenter will find holes suitable for high-resolution cryo-EM in this class. Class 3 (‘thicker’) exhibits a large average vitreous ice thickness of 56nm, which might be slightly too thick. However, due to its broad thickness distribution, it may also contain suitable foil-holes, although fewer. As an additional assessment on ice quality at the particle level, we measured high-resolution cryo-EM images in a class 2 (‘optimal’, Fig. 3d) and in a class 3 (‘thicker’, Fig. 3e) grid square. In the sample image for class 2, the lengthy SbcCD particles are clearly visible individually (white arrows in the insert), and exhibit a density appropriate for imaging. In contrast, the particles visible in the class 3 image are more crowded, forming local aggregates (grey arrow) and exhibit a lower contrast, confirming our classification once again. Finally, in class 4 the contrast is completely lost in EM images (as can be seen from the dark pattern in the center of the rightmost grid square EM image in Fig. 3b), whereas in class 1 the vitreous ice layer is too thin to fit the particles. Additional high-resolution cryo-EM are shown in Supplementary Fig. 5 for classes 1–4. Finally, as an additional control for our classification, we checked if class 1 can also be detected in grids without coated sample. We acquired interferometric images of uncoated copper and gold grids: all detected grid squares (without defects) were identified as class 1 (Supplementary Fig. 6). Thus, our ANN-based analysis pipeline detects and classifies sub-regions of the coated grids reliably as a function of the ice thickness/quality.

### Application to test samples

**Figure 4:**
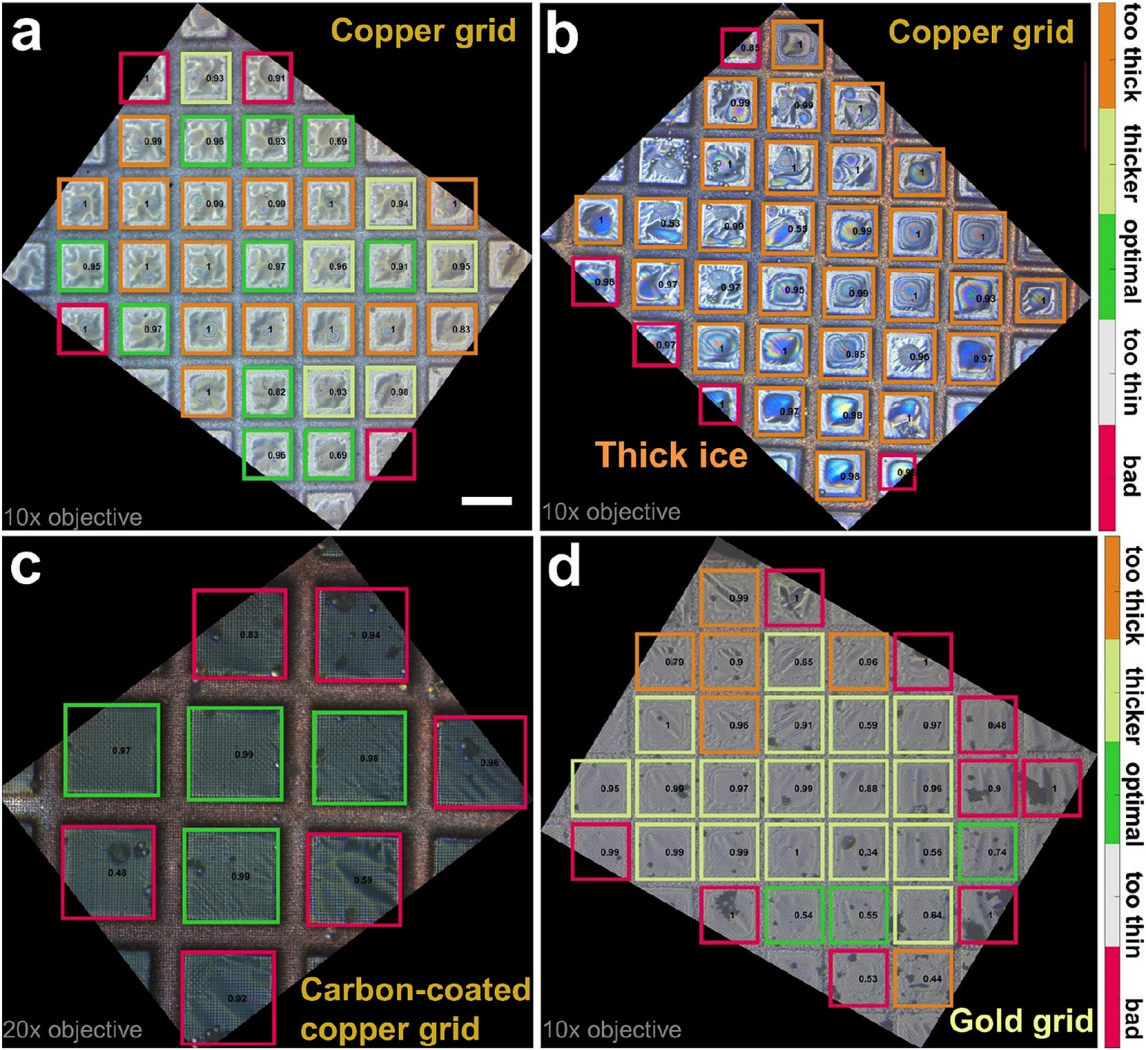
Application to EM grids coated with samples. Examples after having applied our method for copper (**a,b**), carbon-coated copper (**c**), and gold (**d**) grids. While (**a**,**b**,**c**) were imaged using a 10 × magnification objective, (**c**) was measured at higher resolution using a 20 × objective. The individual grid squares are highlighted with colours according to the ice layer quality defined in this work by 5 classes: bad (class 0, in red), too thin (class 1, in white), optimal (class 2, in green), thicker (class 3, in bright green) and too thick (class 4, in red), respectively (colour legends indicated on the right). While the grids shown in (**a)** and (**c**) exhibit a nearly ideal ice behaviour for most of its grid squares (indicated by numerous green and bright-green classified grid squares; the squares in red in (**c**) were only partially imaged), the grid in (**b**) shows only regions with thicker ice (in orange), and the one displayed in (**d**) is an intermediate case with a mixture of class 2 and class 3 grid squares. Numbers indicate confidence of ANN. Scale bar 50 µm.

To apply our method, we collected interferometric images of 20–30 copper and gold grids coated with samples under different conditions, and processed them with ANNICAS (Fig. 4). Whereas most of the grid squares of the copper grid shown in Fig. 4a were identified as class 1 (green squares) and therefore were well-suited for cryo-EM, the second copper grid in Fig. 4b contains too many class 3 grid squares (in orange). Figure 4c shows an higher-magnification image of a carbon-coated copper grid, in which class 2 grid squares were identified (in bright green), potentially containing ‘good’ regions suitable for EM imaging. In the last example, the gold grid (Fig. 4d) contained a mixture of class 2 and 3 objects.

## Discussion

While recent advances in instrumentation have greatly contributed to the development of single particle cryo-EM, sample preparation remains a significant bottleneck in the experimental procedure. We propose a solution for the fast and convenient monitoring of ice thickness using an optical microscope coupled to automated analysis of the ice quality. Our imaging setup is based on standard components with simple optics and moderate costs; it is therefore relatively straightforward to build this setup, requiring only moderate skills in optics, and the microscope is affordable by many research groups.

In our experience it is always necessary to screen several samples before data collection, and the bottleneck for most users is the expensive cryo-EM measurement time. In particular, ice thickness optimization is often a long process requiring multiple trial and error steps between collaborating research groups and EM facilities in an iterative manner. For external users, it is very challenging to prepare grids without immediate quality control. Thus, many samples reaching the facilities from collaborators are of low quality, e.g., exhibiting non-uniform ice, far too thick ice, or no ice at all. By monitoring ice thickness on the coated grids with an easy-to-use and relatively cheap standalone system, our technique can help scientists without a direct and continuous access to an electron microscope to optimize their samples before sending them to facilities. This will save expensive microscope time in the EM facilities. Note that our method is not meant to replace the grid screening procedure at the cryo-EM, since we cannot monitor the particle quality.

A limitation of our approach in his current state is that it does not allow the direct creation of a map with the absolute vitreous ice layer thicknesses across the sample. This could however be achieved by calibrating the height measurements of the ice layer, as was already demonstrated for the 2D imaging of polymer or semi-conductor ultra-thin films^17,20^. However, these structures are more homogeneous than vitreous ice layers on EM grids, for which the calibration of the heights could be more difficult. Due to the strong ice layer heterogeneity including the presence of vitrified ice puddles, it is not possible to conclusively define the ice layer thickness on a given gridsquare. Furthermore, a more complex experimental procedure including thickness calibration is not necessary for our purpose, as the aim of our technique is to assess sample quality rather than the characterization of the absolute thickness profile of the vitreous ice layer. In this work, we propose a different analysis strategy based on ANN that presents the advantage, after proper training, to reliably identify regions of the sample suitable for cryo-EM. We trained our ANN for grid types that are commonly used in the field, but any other grid structure (in other material, grid square size, different coatings, etc.) can be used, provided that the user performs a new training for those specimens. The training results can easily be implemented in ANNICAS by a user with moderate programming skills (Methods). The number of images of individual tiles required for training depends on the number of classes desired and is typically in the order of thousands, corresponding to a few hundreds of interferometric images. However, since it is possible to combine new training data with former ones since EM samples show high similarities, the number of images necessary could even be reduced to a few tens, which makes the image acquisition and the image labelling fast and straightforward. The training process of the two ANNs is critical for the performance of the algorithm. For a few samples (< 10%), the pattern recognition was not sufficient (white arrows in Supplementary Fig. 3a). This could however be further improved by adding more data (especially from different labs) to the training sample.

Finally, our method could be further imporved by enabling the automatic selection of suitable grid squares, that would be located again by a script later, and approached on the electron microscope for imaging at high-resolution. This would require a communication protocol between our ANNICAS and the software controlling the electron microscope. Proprietary issues could be an obstacle with certain commonly used collection suites such as *EPU* (FEI), but open source programs like *SerialEM*^33^ could be adapted. Whereas it is a technically challenging task to integrate the information of the labelled gridsquares in the existing Cryo-EM softwares tools, this operation can be relatively easily performed non-automatically by comparing the interferometric images with the low magnification EM overview images. We illustrate how to correlate the images in Supplementary Fig. 7, and provide in the legend a step-by-step guide for the alignment procedure.

## Conclusion

We have proposed a hardware and software solution for the fast and user-friendly ice thickness monitoring on carbon and gold grids for cryo-EM. Our automated image analysis based on ANN is unbiased, versatile, and can be adapted for various kinds of samples and magnifications. Overall, we believe that this new technique will accelerate the sample optimization procedure, even for scientists who are not expert in EM and laboratories not equipped with an expensive electron microscope. Thereby, it will contribute to facilitate the process of structure determination of protein complexes by cryo-EM.

## Methods

### Sample preparation

Expression and purification of the nuclease deficient H84Q mutant full-length bacterial Mre11 Rad50 complex (EcMR) as well as the *Chaetomium thermophilum* (Ct) Ku70/Ku80 complex was performed as described in Käshammer et al.^34^. The HPLC-purified 120 bp oligonucleotide was purchased from Metabion (Planegg, Germany) and additionally purified via PAGE. For annealing, the complementary oligo mix was heated to 95 °C and then slowly cooled down to 25 °C. Prior to vitrification, the full length EcMR was purified via size exclusion chromatography using a S6 5/150 column in buffer containing 50 mM KCl, 20 mM HEPES at pH 7.5). For the EcMR samples bound to Ct Ku70/Ku80 blocked dsDNA 10 × buffer (10 mM MnCl2, 50 mM MgCl2 and 5 mM ADP-BeF) was mixed with 120 bp dsDNA (fwd:5´GCGTGGCACAACAACTGGCGGGCAAACAGTCGTTGCTGATTGGCGTTGCCACCTCCAGTCTGGCCCTGCACGCGCCGTCGCAAATTGTCGCGGCGATTAAATCTCGCGCCGATCAACTGG-3´) and CtKu70/80 to final concentrations of 0.49 µM and 0.97 µM, respectively. To ensure binding of the CtKu70/80 to the DNA ends, the sample was incubated at 25 °C for 15 min. The size exclusion peak fraction of EcMR was added to a final concentration of 1.47 µM, resulting in a 1:2:3 molar ratio of DNA:CtKu70/80:EcMR. Following EcMR addition, the sample was incubated for additional 15 min at 25 °C and subsequently kept on ice until grid preparation. We applied the protein solution to Quantifoil Cu 200 R2/1 or UltraAU R1.2/1.3grids after glow discharge for 20 s at 20 mA (GloQube, Quorum). Grids were prepared using a *Leica* EM GP plunge freezer (Leica) at 10 °C and 95% humidity. For each prepared grid 4.5 µl of sample was used.

### Single particle cryo-EM

Individual cryo-EM images were collected on a Titan Krios G3 transmission electron microscope (Thermo Fisher Scientific) operated at 300 kV with a K2 Summit direct electron detector and Bioquantum energy filter (Gatan). All images were collected using the EPU software package (Thermo Fisher Scientific) with an applied defocus range of -3.5 µM and a magnified pixel size of 1.059 Å for the largest magnification of 130000x. The lower magnification grid overview and grid square images were collected at 3500- and 8000-fold magnification, respectively.

### Interferometric microscopy setup

Our setup is based on an upright widefield microscope, using a Zeiss *Axiophot* body equipped with three long distance achromatic objectives (Zeiss *Plan-NEOFLUAR 5x/0.16NA Dry*; Zeiss *Plan-NEOFLUAR 10x/0.30NA Dry*, Zeiss *Plan-NEOFLUAR 20x/0.50NA Dry*) and with a mechanical stage on which the cryo-stage (see below) is placed. Note that to have different magnifications available is convenient for more flexibility in the choice of the sample support, but one magnification (we use usually the 10x) is sufficient to screen most of the grid types. The grids are illuminated by a beam from a white-light source that passes through a multi-bandpass filter (*433/517/613 HC Tripleband Filter*, AHF, Germany), and is reflected by a 50/50 beamsplitter (Fig. 1a). The light is then reflected by the sample and, after passing the beamsplitter, is focussed on the chip of a colour camera (Zeiss *AxioCam HRC*) where it produces interference colour. Images are acquired using the software *Axiovision (Rel4.8)* (Zeiss). Note that ANNICAS can run continuously in background and independently from the image acquisition software (see below for details) on the same computer. Thus, any program (such as the open-source suite *MicroManager*^35^) can be used to control the microscopy setup and the camera.

### Cryo-stage

The custom-made cryo-stage consists of a foam dewar (Subangstrom), on which a grid-box transfer mount and an aluminium heat sink are screwed at the bottom. The grid-box transfer mount (arrow 3 in Fig. 1b) comprises two round slotted bolts and a metal cylinder of varying diameter positioned in such a way that the grid-box fits just in between. The metal cylinder can be rotated manually by pushing a screw with a tweezer to fix or to release the grid-box (more details about the design and the fabrication process on demand). While this design is convenient to ensure that the grid-box is tightly fixed, it could however be simplified by screwing a third round slotted bolt instead of the metal cylinder. The aluminium heat sink (Fig. 1c) is made out of an aluminium block with 6 holes drilled with a diameter slightly larger than the one of the grids to accommodate them, and with a depth similar to the grids thickness. Grooves, cut in such a way that they connect the holes (insert in Fig. 3c), ensure that the grids can be conveniently caught by the tweezer.

### Transfer of the grids and imaging procedure

Wearing a mouth plastic shield is strongly recommended during grid manipulation to avoid the formation of frozen water particles due to experimenter breathing. Before use, the cryo-stage must be filled with liquid nitrogen at a slightly higher level than the aluminium heat sink thickness. Importantly, it is necessary to wait for a few minutes until thermic equilibrium is reached. The grid-box can then be placed in the grid-box transfer mount, and the individual grids can be transferred with the tweezer to the holes in the aluminium heat sink as soon as the nitrogen level becomes lower than the height of the holes (after nitrogen evaporation). The samples lie then in a nitrogen gas phase, and are ready for the interferometric imaging. After focussing the illumination beam onto the surface of the grid, different regions of interests (typically 2–3 per grid) or the full grid (by acquiring tiles) can be imaged with exposure times of typically 20-200 ms. The volume of liquid nitrogen present in the cryo-stage is generally sufficient to allow for enough time to image the four grids that can be contained in the grid-box. After image acquisition, the grids are transferred back to the grid-box, that can be stored in a liquid nitrogen container for future use. We checked that this procedure of manipulating and imaging the grids do not damage the vitreous ice layer containing the protein complexes (data not shown).

### ANN-based image analysis

The core of ANNICAS is written in MATLAB^®^ using the *Deep Learning Toolbox™, Image Processing* Toolbox™ (mainly for extracting the tiles before the ANN was trained and for minor post processing after segmentation (see main text), and for the radon transformation), the *Computer Vision Toolbox™*, and the *Parallel Computing* Toolbox™. Although there are augmentation routines in MATLAB^®^, we wrote our own codes to have full control over the augmentation process. During training, the samples were validated after each 4th iteration. For fitting a pretrained network of any architecture, we used the subroutines *createLgraphUsingConnections*, *findLayersToReplace* and *freezeWeights* that are publicly available on *GitHub* (https://github.com/GeneCenterMunich/ANNICAS/tree/master).

For the tile recognition, we only need two classes for segmentation: the tiles and everything else, which is regarded as background (Fig. 2b). The labelling of the tiles was performed manually (using the *MATLAB® ImageLabeller*) in order to achieve highest possible accuracy. We tested all five publicly available (*Deep Learning Toolbox™ *) pre-trained U-shaped ANNs with 650 images and the corresponding pixel labels with all different kinds of grids (copper, carbon-coated copper, and gold), three different image resolutions corresponding to different magnifications of the objectives, and randomly chosen rotation angles (Supplementary Fig. 3a). Furthermore, we augmented all images by rotating them randomly (two copies each with different angles) and by cutting out random regions of random sizes and orientation (again two copies each) in order to simulate different magnifications and points of views. We obtained the best result with *inceptionResNetv2* (stochastic gradient descent^36^, piece wise learning schedule starting at α = 0.001, dropping each 4th iteration by 30%, L2 = 0.005 and momentum 0.9) yielding a mean accuracy for tile recognition of 90% meaning, that 90% of all the pixels are correctly identified as tile or background, respectively. Note, that this accuracy refers to the mean over the entire data set. The vast majority of the grids squares in most images have been recognized almost as good as the labelling (see e.g. Fig. 2b). For some tiles minor post processing with standard image processing tools (filling holes, removing asymmetric bounding boxes) was needed in order to recover detection.

We compiled the source code to a stand-alone executable using the *MATLAB*^®^ Compiler™.

ANNICAS requires a minimum of 3 GB RAM and can be launched via the Microsoft^®^ Windows^®^ command line interface (cmd.ex) with  > *ANNICAS.exe “\path\target_folder\raw_images” “\path\target_folder\analyzed_images”*.

It then calls the networks for segmentation (*inceptionResNetv2*) and classification (*darknet19*) in the memory (approx. 1.8 GB) and waits for a new image to appear (either manually by the user or automatically from the microscope) in the target folder for the raw image. ANNICAS then automatically recognises the new image, performs the analysis, and saves the analysed image in the target folder for the analysed images.

ANNICAS is designed to run continuously in the background (unless getting shut down by the user) to perform automated analysis while using only negligible CPU capacities. Once the software detects a new image it uses available GPU resources to perform the analysis as fast as possible and then turns back to stand by. Since ANNICAS had been compiled from MATLAB^®^ source code a MATLAB^®^ *runtime (version 9.11.)* has to be installed on the computer. The runtime is available free of charge and installation is self-explanatory (https://www.mathworks.com/products/compiler/matlab-runtime.html).

## Supplementary Information

Supplementary Information.

## Data Availability

The ANNICAS software can be downloaded as an executable file (for Windows) at https://github.com/GeneCenterMunich/ANNICAS/tree/master. ANNICAS requires *Matlab runtime 9.11* that can be downloaded free of charge from https://www.mathworks.com/products/compiler/matlab-runtime.html.
