# Supplementary material for: Ice thickness monitoring for cryo-EM grids by interferometry imaging
Source: Sci Rep. 2022 Sep 12;12:15330. doi: 10.1038/s41598-022-16978-7 (PMC9468024; doi:10.1038/s41598-022-16978-7)
Supplement: Supplementary file 1 — Supplementary Information. [file 41598_2022_16978_MOESM1_ESM.docx]

# Supplementary information

**
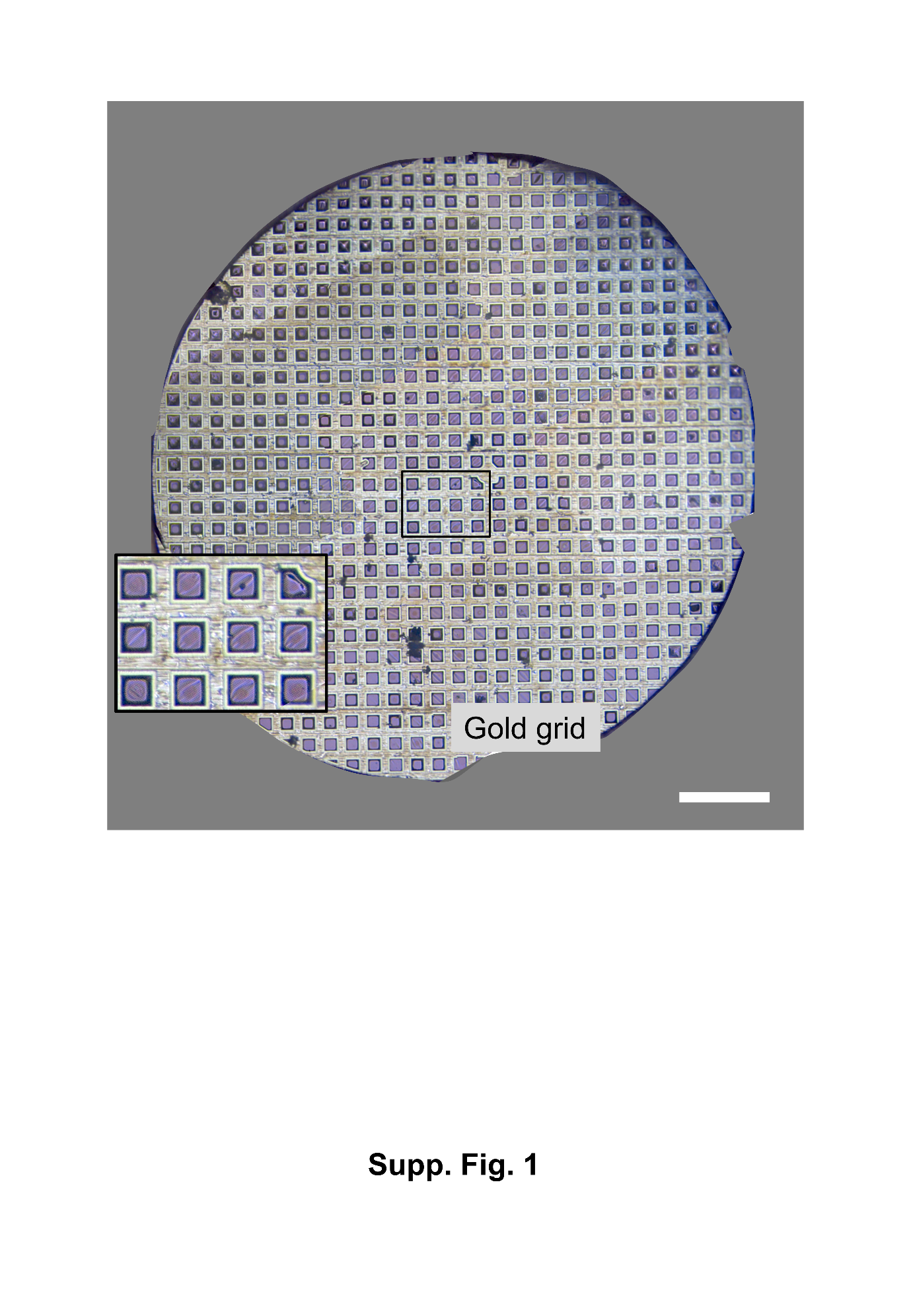
**

**Supplementary Figure 1**. **Optical imaging of a whole grid coated with sample.** Merged interferometric image of a gold grid made of 12 individual tile-images, and acquired by our optical setup with a 10x objective. The insert shows a magnification of the region highlighted in black showing 12 individual grid squares that exhibit colour interference patterns, which correspond to different ice thicknesses/qualities. Scale bar 150 µm.


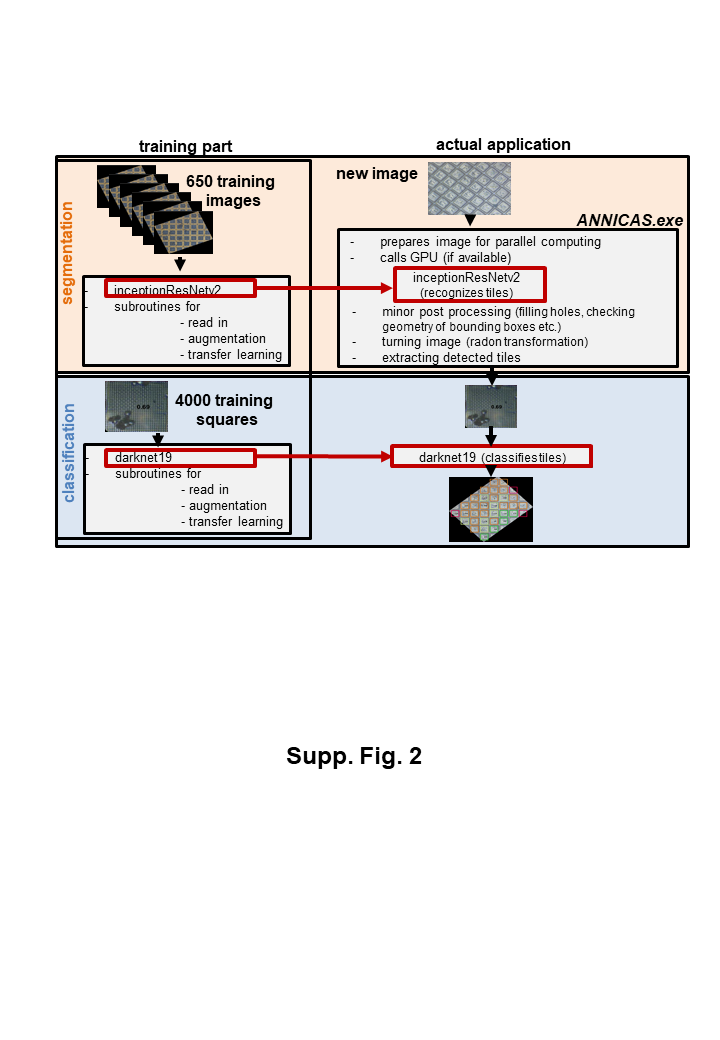


**Supplementary Figure 2**. **Structure of ANNICAS**. We trained the publicly available *inceptionResNetv2* network (upper left) with ca 650 images (containing all kinds of grids and resolutions available) for detection of the grid squares (segmentation) and saved the trained network. In parallel, we trained the *darknet19* (bottom left) network with ~4000 previously labelled grid squares for classification. Both, now trained, networks are called in the ANNICAS executable (right). ANNICAS is launched only once (via CMD) and reads the networks into the memory (1.8 GB, ~1% CPU). Once the microscope generates a new image in the target folder (first input argument of the executable), ANNICAS automatically detects the new image and starts the segmentation analysis (upper right). Since segmentation might not be perfect, minor post processing steps follow. The detected tiles are getting cut out based on their bounding boxes (bottom right) and are classified by calling the trained darkent19 network. Finally, ANNICAS saves the analysed image in a second target folder (second input argument of the executable). Both networks (*inceptionResNetv2* and *darknet19*) are part of the ANNICAS executable and don’t require to be implemented in advance. ANNICAS also automatically runs the analysis on GPUs, if available, to speed up the process.


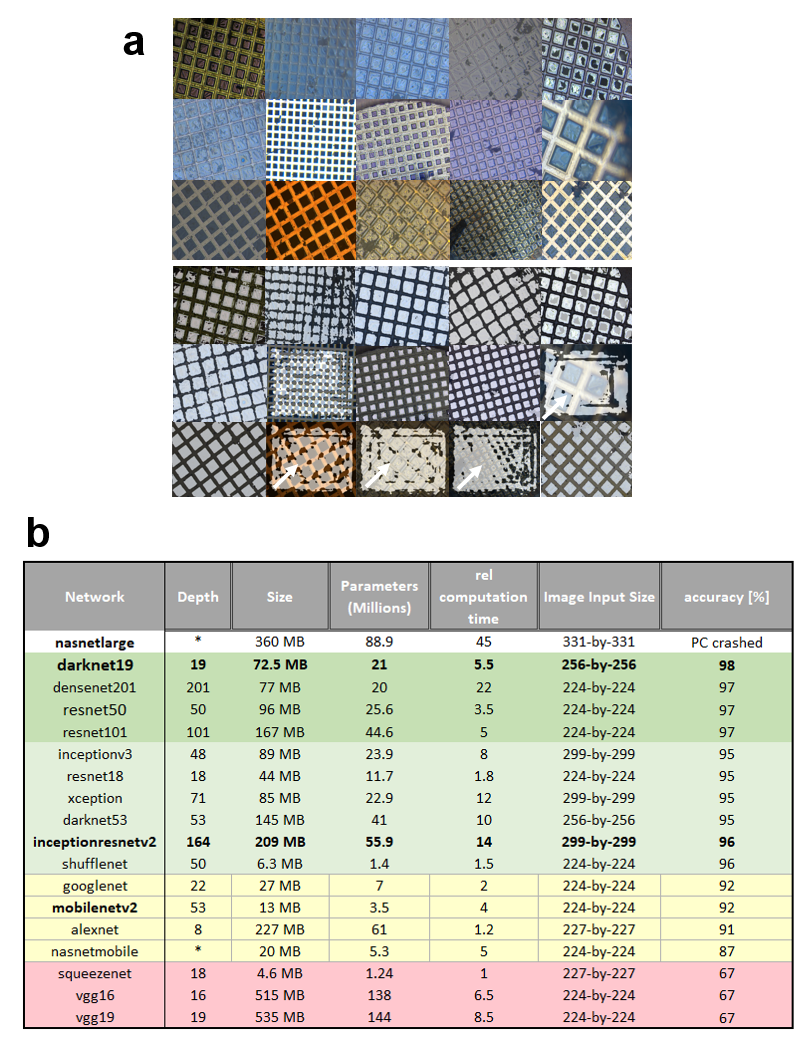


**Supplementary Figure 3**. **Pattern recognition training and labelling for grid square classification.** (**a**) Representative overview over the 650 interferometric images of EM grids acquired using 5x, 10x, and 20x objectives, and with different grids. we used for training the segmentation CNN (left) and how the grid squares were recognized after full training by the network (right). Note, that for some grids the detection is not yet perfect (white arrows). This could however be improved by incorporating additional data. (**b**) List of the 17 publicly available (*deep learning* toolbox in *Matlab*) pretrained CNNs together with their properties and performance after they had been trained with ~4000 tiles.


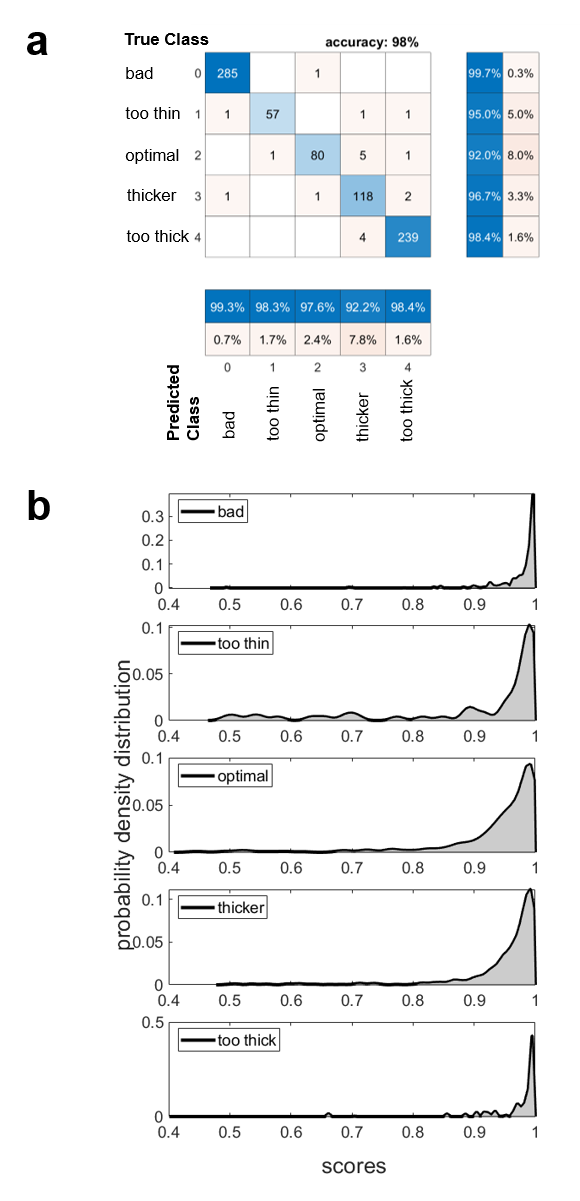


**Supplementary Figure 4**. **Performances of the ANN classification** (a) Confusion chart illustrating the accuracy of the classification ANN throughout the different grid square classes after being trained with ~4000 grid square images (**Methods**). Among the 17 publicly available pre-trained CNNs tested (see main text and **Methods**), the CNN *darknet19* exhibited the best performance with a validation accuracy of 98%. The high score of the validation accuracy demonstrates the accurate classification of the grid square tiles by our training. (**b**) Normalized histograms showing how confident the classification network was for identifying the corresponding classes of the grid squares after being trained with ~4000 grid square images. Ideally, the network is always 100% sure, resulting in a delta function at 1.0. As can be seen, our network is close to the ideal case of minimal cross entropy.


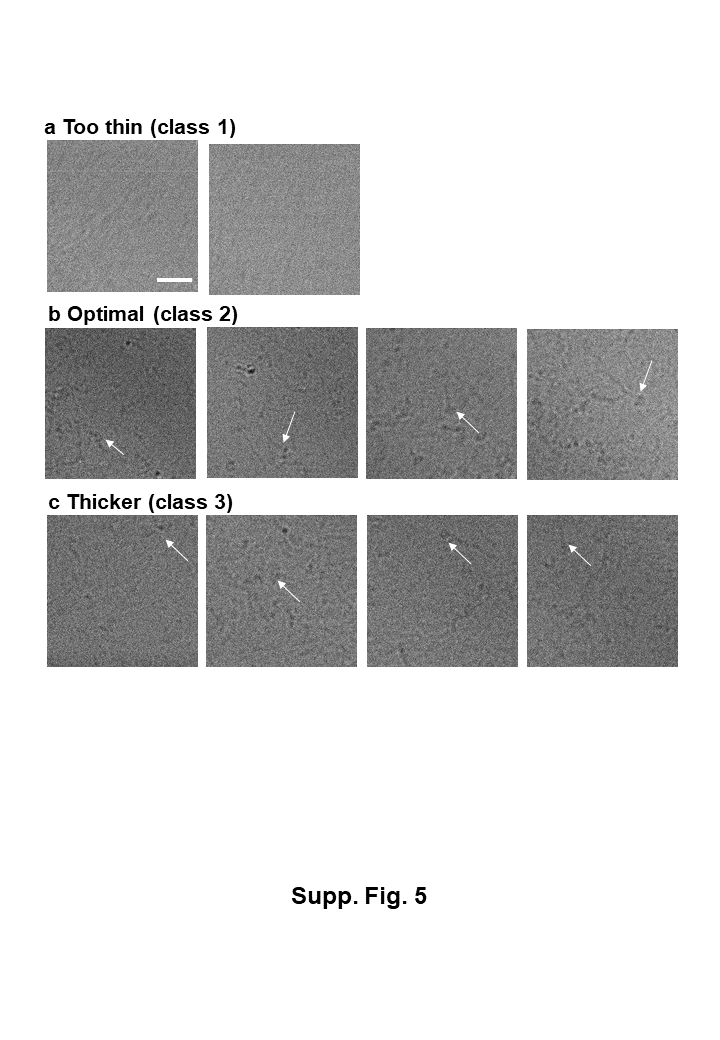


**Supplementary Figure 5**. **High magnification EM images from gridsquares classified as classes 1-3 by the ANN analysis.** Representative EM images (from four different copper grids) of gridsquares classified by our software as (**a**) ‘too thin’ (class1), (**b**) ‘optimal’ (class2), and (**c**) ‘thicker’ (class3) respectively. No SbcCD particles are visible in the ‘too thin’ images (**a**), whereas the ‘optimal’ images (**b**) exhibit many individual particles (examples highlighted with white arrows). This is also the case for the ‘thicker’ class (**c**), however with regions more crowded with particles which also exhibit slightly lower contrast. Scale bar 10 nm.


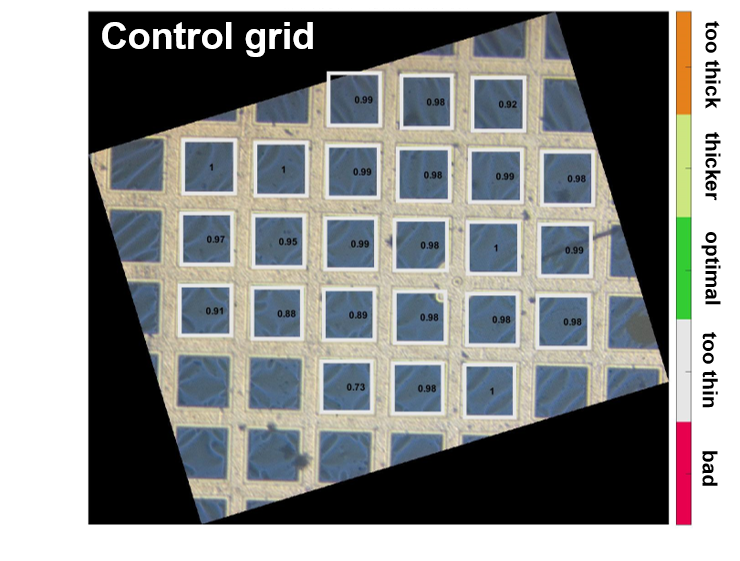


**Supplementary Figure 6**. **Control copper grid without the coating of sample.** The panel displays the interferometric image analyzed by ANNICAS. As expected with absence of a vitreous ice layer, all the grid squares are highlighted in white (‘too thin’). Colour-code for the ice quality shown on the right. Numbers indicate confidence of ANN.


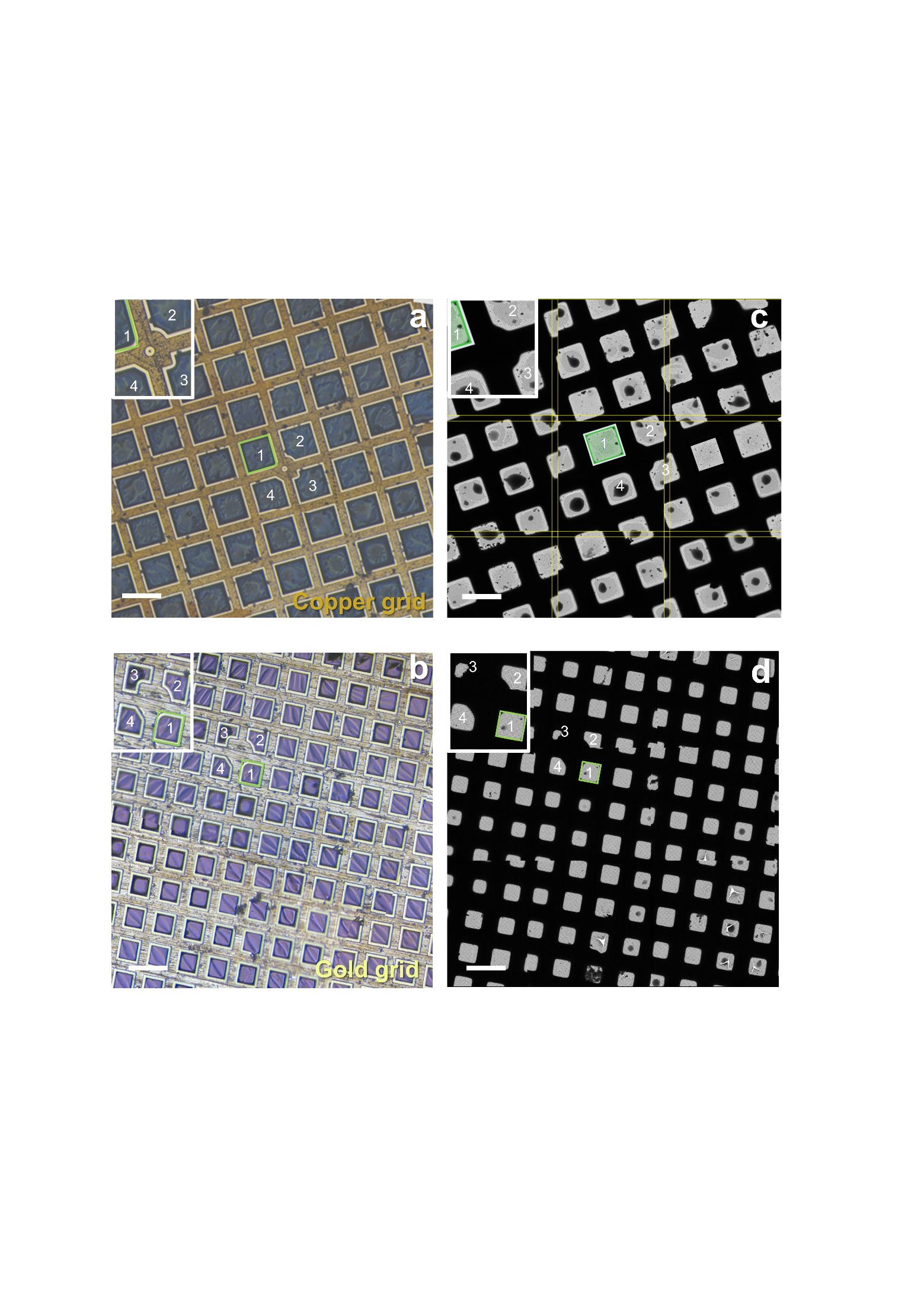


**Supplementary Figure 7**. **Alignment procedure between the interferometric and the EM overview images.** The information of the labelled gridsquares can be transferred to the electron microscope after alignment between the tiled optical images and the low magnification EM images (usually termed “atlas”). The center of standard grids usually exhibits an asymmetric pattern due to the edges of the surrounding gridsquares, which are differently cut on their edges (inserts in **a-d**). This can be conveniently used as a reference for alignment. Alternatively, to use gilder finder grids could facilitate the alignment, although this is not necessary for most available grid types. The alignment procedure consists of the following steps:

- Acquire a series of interferometric images imaging the whole grid by moving manually the XY stage of the optical microscope and ensuring a small overlap between the images of neighbouring regions of the grids. Imaging a grid is usually fast (a few minutes) and requires the acquisition of only 9-12 images using a 10x magnification objective.
- Tile the interferometric images to obtain an overview image of the grid. This operation can be performed manually by using a vector-based drawing software such as *Inkscape* (free of charge, <https://inkscape.org/>), *Illustrator (Adobe)* or *Powerpoint (Microsoft),* or manually with for example the *ImageJ* Grid/Collection Stitching plugin (<https://imagej.net/plugins/grid-collection-stitching>). Examples are shown in **a** and **b** for a copper and a gold grid, respectively.
- Obtain an overview EM image of the grid by using the electron microscope software. Usually, all commercial and non-commercial solutions have implemented a routine that can automatically acquire a series of low magnification images of the grid and tile them into an overview image of the grid (**c** and **d**).
- Identify by visual inspection the middle of the grid in the interferometric and the EM overview images.
- Use the middle of the grids as reference to align the two overview images by translating and rotating e.g. the interferometric overview image using a vector-based drawing software.
- Identify any labelled gridsquares on the EM overview image by counting the number of gridsquares acting as a “coordinate” system in X and Y, starting from the center of the grid.

All these steps can also be performed fully automatically with custom-written script of any programming language. Scale bars 10µm.

REFERENCES:

^1^ Mastronarde, D.N. Automated electron microscope tomography using robust prediction of specimen movements. *J Struct Biol* 152, 36-51 (**2005**).
